# Plasticity of Corticospinal Neural Control after Locomotor Training in Human Spinal Cord Injury

**DOI:** 10.1155/2012/254948

**Published:** 2012-06-04

**Authors:** Maria Knikou

**Affiliations:** ^1^Graduate Center and Physical Therapy Department, College of Staten Island, City University of New York, Staten Island, NY 10314, USA; ^2^Physical Medicine and Rehabilitation, Northwestern University Feinberg School of Medicine, Chicago, IL 60611, USA; ^3^Sensory Motor Performance Program, Rehabilitation Institute of Chicago, Chicago, IL 60611-2654, USA; ^4^Electrophysiological Analysis of Gait and Posture Laboratory, Rehabilitation Institute of Chicago, Chicago, IL 60611, USA

## Abstract

Spinal lesions substantially impair ambulation, occur generally in young and otherwise healthy individuals, and result in devastating effects on quality of life. Restoration of locomotion after damage to the spinal cord is challenging because axons of the damaged neurons do not regenerate spontaneously. Body-weight-supported treadmill training (BWSTT) is a therapeutic approach in which a person with a spinal cord injury (SCI) steps on a motorized treadmill while some body weight is removed through an upper body harness. BWSTT improves temporal gait parameters, muscle activation patterns, and clinical outcome measures in persons with SCI. These changes are likely the result of reorganization that occurs simultaneously in supraspinal and spinal cord neural circuits. This paper will focus on the cortical control of human locomotion and motor output, spinal reflex circuits, and spinal interneuronal circuits and how corticospinal control is reorganized after locomotor training in people with SCI. Based on neurophysiological studies, it is apparent that corticospinal plasticity is involved in restoration of locomotion after training. However, the neural mechanisms underlying restoration of lost voluntary motor function are not well understood and translational neuroscience research is needed so patient-orientated rehabilitation protocols to be developed.

## 1. Introduction

Spinal cord injuries (SCIs) cause substantial social, economic, and health burdens. In the majority of cases, the spinal cord is not completely severed and thus some fiber tracts and segmental spinal cord circuits remain intact [1], which determine the preserved functions and provide the basis for functional restoration. In incomplete SCI persons, recovery of sensorimotor function increases progressively during the first year [2], with reorganization of sensory and motor cortices [3] to lead to recovery of function and maladaptive behavior. In para- and tetraplegic patients, the cortical hand area was expanded towards the cortical leg area and was different based on the lesion level [4]. Further, in paraplegic patients the representation of the nonimpaired upper limb muscles was modified showing an increased activation in the corresponding primary motor cortex (M1), in the parietal cortex, supplementary motor area, and cerebellum [5]. An fMRI study in rats showed that after midthoracic spinal cord transection, deafferented hindlimb territories in S1 exhibited responses to electrical stimulation of the unaffected forepaw, presumably mediated by both spinothalamic and dorsal column nuclei pathways [6]. Evidence suggests that functional plasticity of motor cortical representations is mediated by an anatomical framework of preexisting projections that transverse representation borders [7].

In addition to spontaneous reorganization of the brain after SCI, spinal cord circuitries have the capacity to alter their structure and function with motor training [8], as supported by the physiological leg muscle activation patterns observed after locomotor training in spinalized animals [9–14]. Body weight-supported treadmill training (BWSTT) is a therapeutic approach in which a person with SCI steps on a motorized treadmill while some body weight is removed through an upper body harness [15] and repetitive rhythmic leg movement patterns are promoted either through manual assistance provided by therapists or through a robotic exoskeleton system. Evidence that supports this intervention has been derived largely from studies conducted in spinalized animals [16–19]. Specifically, treadmill training increases axonal regrowth and collateral sprouting proximal to the lesion site in mice [20], phosphorylation of Erk1/2 in the motor cortex as well as the spinal cord injury area [21], expression of brain-derived neurotrophic factor (BDNF) in the spinal cord [22], ameliorates muscle atrophy in moderate contused SCI rats [23], and alters properties of spinal motor neurons [24]. These changes are only a small representation of activity-dependent plasticity located at the synaptic terminals of a variety of systems, that involves physiological, structural, and biochemical changes (see more in [25, 26]).

In humans, BWSTT improves lower extremity motor scores, increases the amplitude of muscle activity in the ankle extensors during the stance phase of walking, and improves walking ability and clinical outcome measures [27–31]. A recent single-blind, randomized clinical trial involving BWSTT with manual assistance, stimulation, over-ground training with stimulation and treadmill training with robotic assistance showed improvements in walking speed and distance [31]. Walking speed was not significantly different between groups, but distance gains were greatest with overground walking training. Further, lower extremity motor scores increased in all groups, regardless the type of intervention [31].

Based on the aforementioned findings, it is apparent that BWSTT contributes to restoration of locomotion. Because remodeling of neuronal circuits as a result of plasticity occurs at multiple sites of the central nervous system [8, 32] restoration of movement after training is anticipated to be the result of neural reorganization that occurs simultaneously in supraspinal and spinal cord circuits. The aim of this paper is to focus on the corticospinal neural plasticity after locomotor training in SCI.

## 2. Cortical Control of Locomotion

The corticospinal tract is the most direct pathway between the cerebral cortex and spinal cord with corticospinal axons monosynaptically synapsing onto spinal motor neurons. Even though neurons of the motor cortex are not required for simple locomotion, they exhibit a profound step-related frequency modulation in the cat [33–35]. This modulation is driven by a combination of signals from the spinal central pattern generators and sensory afferent feedback reflex mechanisms that support interlimb coordination [36]. The modulation of motor cortex neurons is necessary for accurate stepping on uneven terrain when adjustments of the limb trajectory are required to overstep an obstacle or to place the foot on a definite spot on the ground [37–39]. However, pyramidal tract stimulation evokes disynaptic excitatory postsynaptic potentials (EPSPs) in flexor motor neurons that are much bigger in the locomotor state than in the resting state, which are rhythmically modulated so that the facilitation occurs in the flexor-active phase [40]. While the spinal cord of vertebrates possesses the neural structures for genesis of the locomotor rhythm [41–43] and the spinal pattern generator plays a decisive role in the recovery of locomotion after incomplete SCI [12], lesions of the dorsolateral funiculi at Thoracic T13 level in the cat induced long-term deficits on the locomotor pattern [44], supporting that the corticospinal tract plays a prominent role in the neural control of locomotion.

The involvement of supraspinal neural control in human walking can be assessed by a variety of techniques utilized in isolation or in combination, including electroencephalography (EEG), electromyography (EMG), transcranial magnetic and electric stimulation (TMS and TES), and neuroimaging [45, 46]. Single-photon emission tomography and near-infrared spectroscopic topography have shown that the sensorimotor and supplementary motor cortices are activated during real and imagined locomotion [47, 48], while the prefrontal and premotor cortices were involved in adapting the locomotor speed on the treadmill [49]. A recent study postulated a significant coupling between EEG recordings over the leg motor area and EMG from the tibialis anterior (TA) muscle in the frequency band of 24–40 Hz prior to heel strike during the swing phase of walking [50], supporting a cortical involvement in human gait function [51]. (Time (cross-correlation) and frequency (coherence) domain techniques for the detection of coupling between signals provide an analytical framework from which functional coupling between localized cortical activity (measured by MEG or EEG) and motor output (EMG) can be identified in human subjects [52].)

A single stimulus of TMS produces a synchronized discharge of cortical interneurons and pyramidal neurons that travel down the corticospinal tract. Epidural electrodes in the spinal cord detect several waves following TMS, termed direct (D) and indirect (I) waves. I waves originate in the motor cortex most likely through activation of corticocortical projections onto corticospinal neurons [53], while D waves are thought to result from direct depolarization of the initial axon segment of the corticospinal neuron [46]. Recordings from the peripheral muscles demonstrate compound muscle action potentials known as motor evoked potentials (MEPs), which are a summation of multiple motor units depolarizing in response to D and I waves arriving onto the spinal motor neurons [54].

However, the MEP amplitude is not a reliable measure of corticospinal excitability. This is because TMS-induced action potentials in cortical axons spread transynaptically to many other neurons [55] that activate different descending pathways which are differently regulated during human movement [56]. Further, in order to support cortical excitability changes based on alterations of MEP amplitude due to motor plasticity, both need to be mediated by the same motor neurons and caused exclusively by direct monosynaptic projections from the motor cortex without any contamination through indirect interneuronal relays. The peaks in the peristimulus time histogram of the discharge probability of motor units induced by TMS have the same duration as those induced by Ia stimulation, and thus there is ample time for nonmonosynaptic effects to influence the MEP amplitude as is the case for the H-reflex [57, 58]. Lastly, because MEPs are facilitated on average 12 ms before the reaction time to contraction during which antagonists are concomitantly facilitated by subcortical circuits [59, 60], it is apparent that they are sensitive to the excitability state of spinal *α*-motor neurons and interneurons [61].

The aforementioned limitations can be counteracted by reducing the TMS intensity below the MEP threshold. Direct recordings in awake human subjects have shown that TMS at subthreshold MEP intensities, which does not evoke any descending corticospinal volleys, depresses the MEP evoked by a subsequent suprathreshold TMS [62] and the EMG activity of ankle extensor muscles during the stance phase of walking, while the TA ongoing EMG activity is facilitated at a short-latency at early swing phase [63]. At subthreshold TMS intensities the excitability of spinal motor neurons at short latencies is influenced by intracortical inhibitory circuits and mechanisms [62, 64], including but not limited to intracortical and interhemispheric inhibition [65–70], that in turn influence soleus or TA coupled corticomotoneuronal cells. These findings support that cortical excitability changes can be assessed in awake humans and that cortical cells with direct motoneuronal connections change their excitability during human walking. Corticospinal drive of human locomotion is further addressed in Sections 3 and 4, whereas the cortical control of spinal reflex and interneuronal circuits is discussed.

### 2.1. Reorganization of Cortical Control of Motor Output after Training

Various training protocols in uninjured subjects induce reorganization of corticospinal actions on lumbosacral motor neurons. For example, balance training decreased the TA and soleus MEP amplitudes [71], while 32 min voluntary ankle dorsi- and plantar-flexion training increased the TA MEP amplitude regardless of the stimulation intensity level [72]. Repeated visuomotor skill training increased the maximal MEP and decreased the stimulation intensity needed to evoke an MEP, while opposite results were obtained after strength training [73], suggesting that reorganization of corticospinal actions on lumbosacral motor neurons depends on the type of training.

In motor incomplete SCI subjects at rest, MEPs are either absent or very small in amplitude with prolonged latencies, which are considered signs of impaired transmission of the fastest conducting corticospinal neurons [74–77]. Further, the absent or small TA MEPs prevail in SCI persons with increased foot drop [75]. Further, the peak coherence in the 10 to 20 Hz frequency band and synchronization within a narrow time band between paired TA EMG recordings taken during the swing phase were absent during the swing phase and were positively correlated to the degree of foot drop [75]. Because coherence in the frequency and time domain reflects the common synaptic drive, which may be corticospinal in origin, behavioral deficits in ambulatory SCI persons are driven by impaired corticospinal excitability.

Reorganization of corticospinal actions with training in neurological disorders has been shown in few studies. In 4 male SCI subjects with tetraparesis, *f*MRI showed a greater activation in sensorimotor cortical and cerebellar regions following 36 BWSTT sessions [78] consistent with the changes observed in the activation patterns of both hemispheres in poststroke subjects after 4 weeks of BWSTT [79]. Three-to-5 month BWSTT enhanced the MEP amplitude in 9 out of 13 muscles tested, increased the maximal MEP, and changed the slope of the MEP input-output curve in the majority of SCI subjects tested while seated [80]. Furthermore, in incomplete SCI participants whom their locomotor function improved following treadmill training, the coherence (24–40 Hz) of EMG activity, which is thought to indicate a common drive from corticospinal inputs, between antagonist muscles acting at the knee joint was increased and remained unaltered in participants that the locomotor ability was not improved [81]. The lower-frequency coherence (5–18 Hz), which is thought to contain common synaptic drive from spinal inputs, remained unchanged in both groups [81].

**Figure 1:**
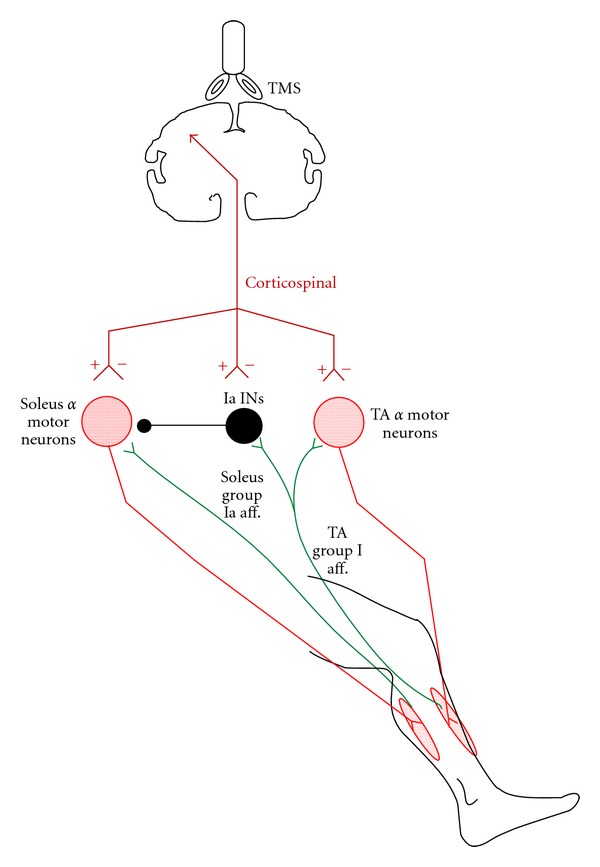
Schematic illustration of cortical control of spinal reflex circuits and spinal interneuronal circuits investigated after 60 sessions of locomotor training in the same SCI subject. The soleus H-reflex evoked by posterior tibial nerve stimulation, tibialis anterior (TA) muscle motor evoked potential (MEP), soleus H-reflex conditioned by subthreshold transcranial magnetic stimulation (TMS) delivered at an optimal site (“hot spot”) for evoking an MEP in the right soleus muscle, soleus H-reflex depression by common peroneal nerve stimulation that is mediated by Ia inhibitory interneurons (Ia INs; reciprocal inhibition), and the reciprocal inhibition conditioned by subthreshold TMS delivered at an optimal site (“hot spot”) for evoking an MEP in the right TA muscle were all investigated in the same patient at rest and/or during assisted stepping after locomotor training. Open triangles indicate excitatory synapses, while the filled circle indicates inhibitory synapses. The cortical control on these spinal circuits is indicated as a synapse that may increase (+) or decrease (−) actions of flexor-extensor *α* motor neurons and/or Ia inhibitory interneurons.

One person (49 yo female, 5 years after-injury) with an American Spinal Injury Association (ASIA) Impairment Scale (AIS) D at Thoracic 5–7 received 60 BWSTT sessions (1 h/day; 5 days/week) with a robotic exoskeleton device (Lokomat). Before training, the patient stepped at 0.5 m/s with 50% body weight support (BWS), and after training the patient stepped at 0.89 m/s with 20% BWS. Electrophysiological tests, illustrated as a schema in Figure 1, were conducted before and after training in the same patient while seated as well as during BWS assisted stepping. Data presented in this paper are original, have not been published elsewhere, and are from the same patient. Experiments and training were conducted following the written consent of the subject. All experimental procedures were approved by the Institutional Review Board of the Northwestern University IRB committee and were conducted in compliance with the 1964 Declaration of Helsinki.

**Figure 2:**
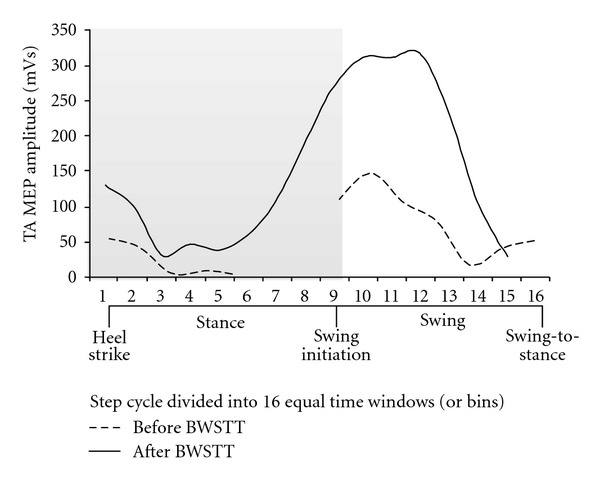
TA MEP modulation during stepping before and after locomotor training in SCI. The tibialis anterior (TA) motor evoked potential (MEP) amplitude before (dashed line) and after (solid line) 60 body weight-supported treadmill training (BWSTT) sessions is indicated as a function of the step cycle during body weight-supported (BWS) assisted stepping for one patient with American Spinal Injury Association (ASIA) Impairment Scale (AIS) D (49 yo female, 5 years after injury, T5–7). The TA MEP was evoked randomly every 3 to 4 steps at 1.3 times TA MEP threshold while stepping at 0.5 m/s with 50% BWS before training and at 0.89 m/s with 20% BWS after training. MEP threshold was established with subject standing at equivalent levels of BWS utilized during stepping. The step cycle was divided into 16 equal time windows or bins. Stance phase duration is identified by the grey region. Bin 1 corresponds to heel strike. Bins 8, 9, and 16 correspond approximately to stance-to-swing transition, swing phase initiation, and swing-to-stance transition, respectively.

The TA MEPs evoked at 1.3 TA MEP threshold during assisted stepping before and after training are shown in Figure 2. (The TA MEP threshold was established with the subject standing at equivalent BWS levels to that utilized during stepping. During stepping, TA MEPs were evoked randomly at different phases of the step cycle every 3 to 5 steps based on the signal from the ipsilateral foot switch. The step cycle of the right leg was divided into 16 equal time windows or bins.) Before training, the TA MEP amplitude was increased during early swing (bins 10–13) when compared to that observed at midstance (bins 3–5), but an MEP was not evocable from mid stance (bin 6) until swing phase initiation (bin 9). After training, the TA MEP amplitude increased significantly compared to that observed before training and was modulated in a phase-dependent pattern; that is, it was progressively depressed during the stance phase (bins 1–7) and was facilitated during the swing phase (bins 9–14) (Figure 2). This TA MEP modulation pattern during assisted stepping is consistent with that reported in uninjured subjects, which is generally increased when the muscle from which it is recorded is active and small when the antagonist muscle is active [82–84]. Although the findings reported in Figure 2 are from a single case, the altered MEP modulation pattern supports the notion that locomotor training alters the efficacy of corticospinal descending motor volleys synapsing with TA spinal motor neurons in a manner that supports a physiologic gait pattern. It is apparent that more studies are needed on the neuronal mechanisms mediating improvement of  locomotor function after training in spinal lesions of different segmental levels and types, in order that currently available rehabilitation strategies are optimized.

## 3. Cortical Control of Spinal Reflex Circuits

The spinal cord constitutes the final common pathway for segmental and supraspinal pathways underlying motor behavior. Electrical stimulation of a mixed peripheral nerve at low intensities activates primary (Ia) afferent axons which synapse in the spinal cord. Alpha motor neurons activated monosynaptically by Ia afferent volleys induce a synchronized reflex response known as the Hoffmann-(H-) reflex [85], which is the electrical analogue of the monosynaptic stretch reflex. When the H-reflex is used as a test reflex, the effects of conditioning volleys from other afferents or descending tracts on the motoneuron pool and synaptic actions from different sources in health and disease can be assessed [85, 86].

Cortical control of spinal reflex circuits has been extensively investigated in awake humans by means of TMS. Subthreshold TMS produces a short-latency inhibition on the soleus H-reflex followed by a period of facilitation [56, 87–89] with subjects at rest. In contrast, the TA H-reflex is facilitated at an early conditioning-test (C-T) interval [87]. Superficial peroneal or sural nerve stimulation potentiates the presumably monosynaptic facilitation of the TA H-reflex evoked by brain stimulation [90]. The cortical modulation of the soleus H-reflex depends largely on the position of the ankle joint, with subthreshold TMS to induce an early long-lasting facilitation or depression of the soleus H-reflex during tonic plantar flexion and dorsiflexion, respectively [87, 91]. Similar findings have been reported for pyramidal monkeys, cats, and baboons during which cortical inhibition predominated on the soleus and gastrocnemius monosynaptic reflex, while cortical facilitation influenced largely flexor motor neurons [92, 93]. It should be noted, however, that a single cortical D wave could produce changes in segmental motor neurons in the primates but not in the cat that required D and I waves or multiple D-waves [93].

In addition to the H-reflex, the TA long-latency (or M3) ankle stretch reflex was facilitated when the MEP arrived in the spinal cord at the same time [94]. However, subthreshold TMS intensities delivered 55–85 ms prior to the M3 depressed the long-latency TA stretch reflex [95]. Because the long-latency response was reduced in size following subthreshold TMS while the short-latency response remained unchanged [95, 96], it provides evidence that the long-latency stretch reflex is mediated in part by a transcortical path that can be affected by subthreshold TMS. During human walking, subthreshold TMS induces a short-latency, presumably monosynaptic, facilitation of the soleus H-reflex followed by a long-lasting inhibition [94]. Because potentiation of TA MEPs was synchronized with the peak TA ankle stretch reflex, corticospinal pathways are partly involved in the generation of spinal stretch reflexes during human walking [97, 98]. In human SCI, the conditioned H-reflex profile by subthreshold TMS varied significantly based on the AIS scores [99, 100]. In patients with severe paralysis (AIS A-B) an early or late soleus H-reflex facilitation by TMS was absent [99], suggesting for a nonphysiological interaction between descending inputs and spinal reflex excitability in patients with spastic paraparesis [100].

### 3.1. Reorganization of Cortical Control of Spinal Reflexes after Training

Persistent changes in H- or stretch reflex amplitudes may be regarded as signs of learning and plasticity as a result of training, which have been shown after various training protocols. For example, 30 min ankle cocontraction training decreased the ratio of maximal H-reflex versus maximal M wave (Hmax/Mmax) and improved motor performance defined as the difference between the maximum and minimum torque displacements within 1 min [101]. The soleus H-reflex amplitude was enhanced after 3 week isometric maximal plantar flexion training when measured at 20% and 60% of maximal voluntary contraction (MVC) [102], with similar results to be reported after 14 week of resistance training that involved heavy weight-lifting exercises for the leg muscles with reflexes measured during maximal isometric ramp contractions [103]. In contrast, 18 sessions eccentric strength training of the plantar flexor muscles for a 7 week period increased the Hmax/Mmax ratio during eccentric MVC but not during isometric or concentric contractions [104], suggesting that spinal reflex excitability is adjusted based on the type of exercise training protocol.

Nonetheless, the aforementioned changes in H-reflex amplitude can result from modifications of interneuronal circuits interposed in the spinal pathway or by changes on the strength of descending pathways, since the latter is potent regulator of spinal reflex circuits behavior [105–107]. This is supported by the failed operant conditioning of the H-reflex in rats when the corticospinal tract was transected at the spinal cord level [108, 109].

Limited evidence exists on plastic changes of the cortical control of spinal reflexes after locomotor training in neurological disorders. Forty BWSTT sessions in 29 patients with incomplete SCI reestablished the TMS-induced long-latency soleus H-reflex facilitation with subjects at rest [110]. It should be noted that BWS improves the efficacy of the sensorimotor cortex function [111], decreases the TA MEP threshold, and increases the map size for the TA in both hemispheres of stroke patients [112]. Nonetheless, when TMS effects on spinal reflexes are assessed with patients at a resting state, it cannot be assumed that corticospinal changes due to training are transferrable at a locomotor state and thus be functional relevant. This is largely based on that (1) short-latency ankle or quadriceps extensor reflexes (H- or stretch reflexes) are modulated in a phase-dependent pattern in uninjured subjects [113–115], (2) the phase-dependent modulation of these reflexes is affected substantially in individuals with an SCI [115–117], and (3) cortical control constitutes one of the sources for the phasic patterned reflex excitability during human walking [86].

**Figure 3:**
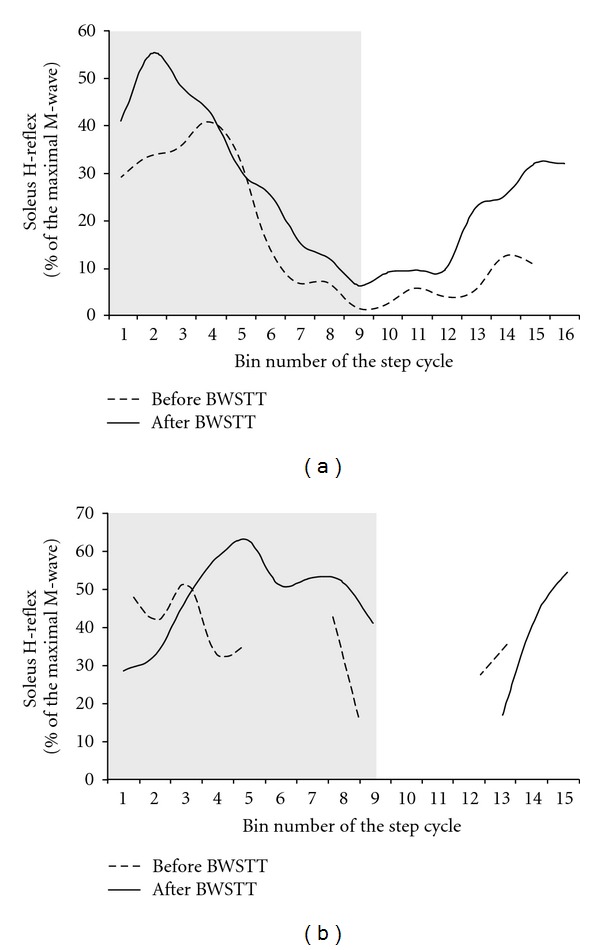
Soleus H-reflex modulation during assisted stepping before and after locomotor training in SCI. The unconditioned soleus H-reflex modulation before (dashed lines) and after (solid lines) 60 sessions of body weight-supported treadmill training (BWSTT) (a) and the conditioned soleus H-reflex by subthreshold TMS at the conditioning-test interval of 1 ms (b) are indicated as a function of the step cycle. The mean amplitude of the unconditioned and conditioned soleus H-reflexes evoked at each bin is expressed as a percentage of the maximal M-wave evoked 80 ms after the test H-reflex. TMS was delivered at 0.95 times MEP threshold for the soleus muscle at a conditioning-test interval of 1-ms. Unconditioned and conditioned soleus H-reflexes were accepted when the associated M-waves ranged from 4 to 8% of the maximal M-wave evoked at each bin. H-reflex values are not indicated for some of the bins after BWSTT because they were not accepted based on the M-wave amplitude as a percentage of the maximal M-wave, which is different from not being evocable as was the case for before BWSTT. The step cycle was divided into 16 equal time windows or bins. Stance phase duration is identified by the grey region. Bin 1 corresponds to heel strike. Bins 8, 9, and 16 correspond approximately to stance-to-swing transition, swing phase initiation, and swing-to-stance transition, respectively.

In Figure 3(a), the soleus H-reflex recorded during BWS assisted stepping according to methods described in detail [115, 118, 119], before and after 60 BWSTT sessions, is indicated for the same patient whose TA MEP modulation pattern was described in Figure 2. After 60 BWSTT sessions, the maximal reflex excitability shifted, with respect to the step cycle phase, from mid- to early stance (bins 1–3), while a maintained H-reflex excitability commonly observed throughout the stance phase in uninjured subjects [115] was absent before and after training (Figure 3(a)). However, after 60 BWSTT sessions the soleus H-reflex amplitude increased during the late swing phase (bins 12–16), consistent to a reflex behavior observed in some control subjects [120]. The effects of subthreshold TMS on the soleus H-reflex at a C-T interval of 1-ms during BWS assisted stepping are indicated as a function of the step cycle before and after 60 BWSTT sessions in Figure 3(b). It is clear that, after 60 BWSTT sessions, subthreshold TMS affected substantially the soleus H-reflex during the stance phase resulting in a progressive increase of the soleus H-reflex amplitude. The soleus H-reflex amplitude was maintained throughout the stance phase (compare bins 1–8 in Figures 3(a) and 3(b)). Modifications in synaptic actions of cortical inhibitory circuits exerted on soleus motor neurons might be the source of these changes since the phasic soleus H-reflex excitability during BWS assisted stepping with or without leg assistance by a robotic exoskeleton remains unaltered [115, 118].

## 4. Spinal Interneuronal Inhibitory Circuits: Reciprocal Ia Inhibition

One of the spinal interneuronal circuits with paramount contribution to the neural control of movement is that of disynaptic reciprocal Ia inhibition. Reciprocal inhibition refers to an automatic antagonist motor neuron inhibition when an agonist muscle contracts. Following an SCI, the reciprocal inhibition is either reduced or replaced by reciprocal facilitation [121–124] leading to coactivation of antagonist ankle muscles, spasticity, and poor movement performance.

Regulation of locomotion by reflexly mediated spinal circuits that integrate sensory inputs is well established. The contribution of muscle afferents mediating information about the amplitude and rate of muscle stretch is easily recognized by the phase-dependent modulation of short-latency spinal reflexes during walking. The short-latency soleus and quadriceps extensor reflexes (stretch, tendon, or H-reflex) in humans are modulated in a way that promotes bipedal gait. The ankle stretch and soleus H-reflexes increase progressively from mid- to late stance in parallel with the soleus EMG activity and are significantly depressed or abolished during the swing phase of gait [113–115, 125]. A phase-dependent modulation has been demonstrated for the ankle stretch reflex in the high decerebrate mesencephalic cat [126].

The soleus H-reflex depression during the swing phase in humans has been partly ascribed to reciprocal Ia inhibition exerted from common peroneal nerve group I afferents on soleus motor neurons, which is regulated in a similar manner to that reported in animals and corresponds largely to absent reciprocal inhibition in the stance phase and maximal in the swing phase [127, 128]. During fictive locomotion in cats without brainstem connections, simultaneous extracellular recordings from Ia inhibitory interneurons and intracellular recordings from lumbar motor neurons revealed that hyperpolarization of soleus *α* motor neurons coincided with activity of Ia inhibitory interneurons [129, 130]. Ia inhibitory interneurons were rhythmically active due to periodic excitation and not due to periodic inhibition by other spinal inhibitory interneurons [130]. Recent evidence obtained from spinalized animals verified that reciprocal Ia inhibition contributes to hyperpolarization of motor neurons during the inactive (flexion) phase of locomotion [131].

### 4.1. Cortical Control of Reciprocal Ia Inhibition

Animal studies through intracellular recordings provided a detailed knowledge of the pathway and integration of segmental and supraspinal convergence at the interneuronal level [132–135] with volleys in the corticospinal tract to exert an excitatory effect over Ia inhibitory interneurons [136]. In monkeys, intracortical stimulation revealed that the same interneurons mediate the disynaptic inhibition of motor neurons evoked by corticospinal fibers and the disynaptic inhibition of motor neurons evoked by group Ia afferents of antagonist muscles [137]. Further, motor neurons and Ia inhibitory interneurons were activated in parallel by supraspinal centers in order to secure a coordinated contraction of agonists and relaxation of antagonists [138, 139].

Descending control of reciprocal inhibition has clearly been postulated in humans. In particular, the reciprocal inhibition exerted from common peroneal nerve group I afferents on soleus motor neurons was observed 50 ms before the onset of TA EMG activity [140]. Further, when subjects attempted to dorsiflex the ankle after the common peroneal nerve was blocked with a local anesthetic a strong soleus H-reflex depression was still evident [141]. The test H-reflex facilitation, induced by TES applied to the scalp below the intensity needed to produce a motor response, was quickly terminated by subsequent arrivals of IPSPs at the motor neurons [88]. These IPSPs might be produced by activity in Ia inhibitory interneurons, which in monkeys receive monosynaptic tract projections [142]. Single subthreshold TES reduced the inhibition of the flexor carpi radialis H-reflex evoked by radial nerve stimulation at a latency compatible with a monosynaptic or disynaptic corticospinal projection to Ia inhibitory interneurons [143]. Descending facilitation of Ia inhibitory interneurons has also been documented for the human leg [87, 89].

### 4.2. Reorganization of Cortical Control of Reciprocal Ia Inhibition after Training

Findings on the reorganization of reciprocal inhibition as a result of motor training in health and disease are limited. Stimulation of the common peroneal nerve with a train of 10 pulses at 100 Hz with and without motor cortex stimulation potentiated reciprocal inhibition in control subjects [144]. Reciprocal inhibition was potentiated after 12 sessions of ankle dorsiflexion strength training when measured at the onset of ankle dorsiflexion but remained unchanged when measured with subjects at rest [145].

**Figure 4:**
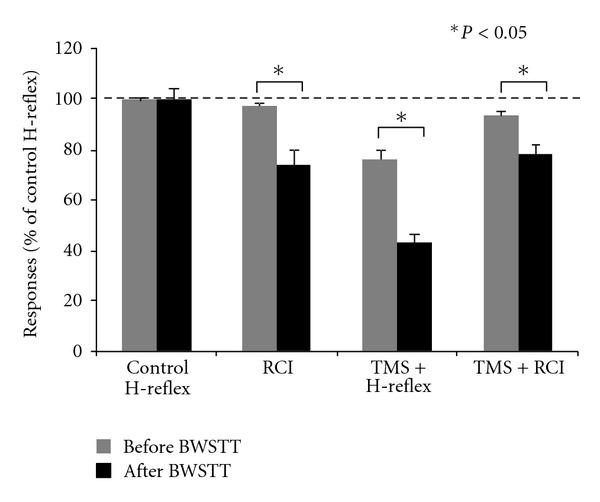
Cortical control of spinal reflex circuits after locomotor training in SCI. Mean size (*n* = 20) of soleus H-reflex conditioned by common peroneal nerve stimulation at the conditioning-test interval of 3 ms, which reflects the amount of reciprocal Ia inhibition (RCI), soleus H-reflex conditioned by subthreshold TMS (TMS + H-reflex) at a C-T interval of 1 ms, and reciprocal inhibition conditioned with subthreshold TMS (TMS + RCI) at C-T intervals of 1 and 3 ms, respectively. Data are from the same patient. The size of the conditioned H-reflexes is expressed as a percentage of the mean amplitude of the control soleus H-reflex. Error bars indicate the SEM, and asterisks denote a statistically significant difference (paired *t*-test, *P* < 0.05) for conditioned H-reflexes recorded before and after 60 BWSTT sessions.

In Figure 4, the mean amplitude of the soleus H-reflex conditioned by stimulation of common peroneal nerve group I afferents at a C-T interval of 3 ms and established according to methods outlined in detail [146], which represents the amount of reciprocal inhibition (RCI), before and after 60 BWSTT sessions with subject seated (same patient for data previously described in Figures 2 and 3 is indicated as a percentage of the control H-reflex). Further, the soleus H-reflex conditioned by subthreshold TMS at a C-T interval of 1 ms and the reciprocal inhibition conditioned by subthreshold TMS (C-T intervals: 3 and 1 ms, resp.) as a percentage of the control H-reflex is indicated. It is apparent that locomotor training reestablished the reciprocal inhibition exerted from flexor group I afferents on soleus motor neurons, potentiated the soleus H-reflex depression following subthreshold TMS, and potentiated the reciprocal inhibition conditioned by subthreshold TMS, consistent with findings reported in uninjured subjects (see Figure 4 in [147]).

**Figure 5:**
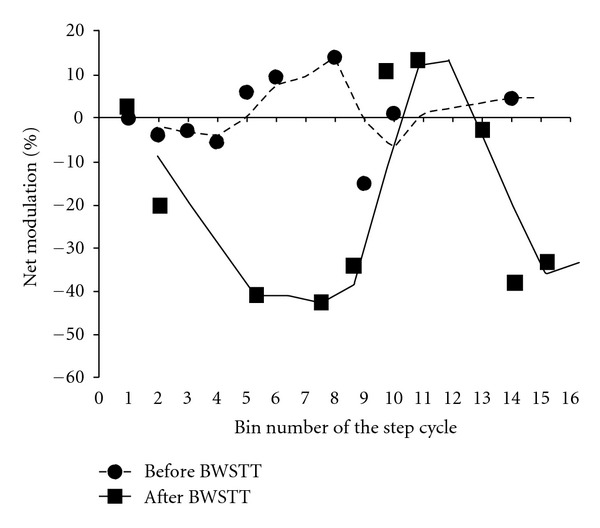
Changes in the cortical control of reciprocal Ia inhibition after locomotor training in SCI. Net effects of subthreshold transcranial magnetic stimulation (TMS) on reciprocal Ia inhibition during BWS assisted stepping before and after 60 body weight-supported treadmill training (BWSTT) sessions. The net effects of subthreshold TMS on reciprocal inhibition were estimated at each bin of the step cycle based on the equation (*D*-*C*)-(*B*-*A*) whereas *A* is the test H-reflex during stepping, *B* is the soleus H-reflex conditioned by subthreshold TMS during stepping at a conditioning-test (C-T) interval of 1 ms, *C* is the soleus H-reflex conditioned by common peroneal nerve stimulation (i.e., reciprocal inhibition) at a C-T interval of 3 ms during stepping, and *D* is the reciprocal inhibition conditioned by subthreshold TMS (3 and 1 ms C-T intervals). Positive values indicate potentiation of reciprocal inhibition and negative values indicate attenuation of reciprocal inhibition by cortical inputs.

The net effects of subthreshold TMS on the reciprocal inhibition during BWS-assisted stepping before and after 60 BWSTT sessions are indicated in Figure 5 for the same patient. The net effects (or net modulation) were estimated at each bin of the step cycle based on the equation (*D*-*C*)-(*B*-*A*) whereas *A* is the test soleus H-reflex (baseline soleus H-reflex modulation pattern during stepping), *B* is the soleus H-reflex conditioned by subthreshold TMS, *C* is the soleus H-reflex conditioned by common peroneal nerve stimulation (i.e., reciprocal inhibition), and *D* is the reciprocal inhibition conditioned by subthreshold TMS. Positive values indicate potentiation of reciprocal inhibition and negative values indicate attenuation of reciprocal inhibition. Locomotor training contributed significantly to attenuation of reciprocal inhibition exerted from ankle flexor afferents to extensor motor neurons during the stance phase. Most importantly, potentiation of reciprocal inhibition at swing phase initiation (i.e., bin 9 in Figure 5) was evident. Adaptation of cortical control of reciprocal inhibition after locomotor training supports that changes of corticospinal neuronal pathways interacting with Ia interneurons are possible in people with a chronic SCI, although altered corticospinal interactions with other spinal inhibitory interneurons, such as Renshaw cells and presynaptic inhibitory interneurons, cannot be excluded [85, 148, 149].

## 5. Conclusion

SCI changes the human body homeostasis leading to myriad changes of multiple systems. In most cases, the spinal cord is not completely severed and thus some fiber tracts and segmental spinal cord circuits remain intact. Based on the plastic capabilities of the central nervous system, it is apparent that the adult lesioned motor system reorganization occurs spontaneously after an injury and after training. Electrophysiological studies have shown that BWSTT increases the MEP amplitude, changes the common drive of antagonist muscles from corticospinal inputs with subjects seated, and alters the TA MEP modulation pattern during BWS assisted stepping. Further, BWSTT reestablished the TMS-induced long-latency soleus H-reflex facilitation and potentiated the short-latency soleus H-reflex depression following subthreshold TMS with subjects at rest, while cortical modulation of the soleus H-reflex during stepping changed significantly. Lastly, BWSTT changed the cortical control of reciprocal inhibition during BWS assisted stepping in a manner that promotes bipedal gait. These findings support the notion that improvements in locomotor function from treadmill training are mediated, in part, by changes in the corticospinal drive of spinal reflex circuits, spinal interneuronal circuits, and output of leg muscles during walking.

## 6. Perspective

Plasticity in the brain and spinal cord underlying restoration of lost function can be driven by appropriately designed interventions [150, 151]. Development of such interventions depends largely on gaining a detailed understanding of the underlying neural mechanisms that support restoration of motor function. Based on this brief paper it is clear that there is a need for translational neuroscience research in order that the neural mechanisms underlying restoration of lost voluntary motor function are outlined based on specific clinical cases. This body of knowledge will contribute significantly to the development of new rehabilitation strategies and/or optimization of the currently available strategies, and to patient-orientated rehabilitation protocols promoting evidence-based rehabilitation.

## Abbreviations

BWS: Body weight support
BWSTT: Body weight-supported treadmill training
C-T: Conditioning-test
EMG: Electromyographic
EPSPs: Excitatory postsynaptic potentials
IPSPs: Inhibitory postsynaptic potentials
MEP: Motor evoked potentials
SCI: Spinal cord injury
TA: Tibialis anterior
TMS: Transcranial magnetic stimulation
TES: Transcranial electric stimulation.
